# Pathogenicity and Metabolomic Characterization of *Fusarium graminearum* and *Fusarium poae* Challenge in Barley under Controlled Conditions

**DOI:** 10.3390/jof10100670

**Published:** 2024-09-26

**Authors:** Raja Khanal, Kerin Hudson, Adam Foster, Xiben Wang, Elizabeth K. Brauer, Thomas E. Witte, David P. Overy

**Affiliations:** 1 Ottawa Research and Development Centre, Agriculture and Agri-Food Canada, Ottawa, ON K1A 0C6, Canada; kerin.hudson@agr.gc.ca (K.H.); elizabeth.brauer@agr.gc.ca (E.K.B.); david.overy@agr.gc.ca (D.P.O.); 2 Charlottetown Research and Development Centre, Agriculture and Agri-Food Canada, Charlottetown, PE C1A 4N6, Canada; adam.foster@agr.gc.ca; 3 Morden Research and Development Centre, Agriculture and Agri-Food Canada, Morden, MB R6M 1Y5, Canada; xiben.wang@agr.gc.ca; 4 Department of Biology, University of Ottawa, Ottawa, ON K1N 6N5, Canada

**Keywords:** Fusarium head blight, *Fusarium graminearum*, *Fusarium poae*, barley, deoxynivalenol

## Abstract

Barley is the third most important cereal crop in terms of production in Canada, and Fusarium head blight (FHB) is one of the main fungal diseases of barley. FHB is caused by a species complex of Fusaria, of which *Fusarium graminearum* Schwabe is the main causal species of FHB epidemics in Canada. Field surveys show that two or more *Fusarium* species often co-exist within the same field or grain sample, and *F. poae* is reported as another important species in barley. This study aimed to determine the pathogenicity of *F. graminearum*, *F. poae*, and a co-inoculation of both species causing FHB in barley. Two susceptible barley cultivars were spray-inoculated at 10 to 14 days after heading. Phenotypic disease severity was rated on a scale of 0–9 at 4, 7, 14, 21, and 28 days after inoculation. There was a significant difference in FHB severity between *F. graminearum* and *F. poae*, where infection with *F. graminearum* produced more severe disease ratings. *F. poae* generated lower disease ratings and was not statistically different from the control. When heads were co-inoculated with both *Fusarium* species, the resulting FHB severity was unchanged relative to heads inoculated with *F. graminearum* only. The ratio of *F. graminearum* to *F. poae* genomic DNA was also no different than when heads were inoculated with *F. graminearum* alone, as quantified with ddPCR using markers specific to each species. The metabolomic analysis of sample extracts showed that *F. graminearum*-associated metabolites dominated the mycotoxin profile of co-inoculated samples, which corroborated our other findings where *F. graminearum* appeared to outcompete *F. poae* in barley. No significant effect on visual FHB disease ratings or fungal DNA detection was observed between the cultivars tested. However, there were some metabolome differences between cultivars in response to the challenge by both *F. graminearum* and *F. poae*.

## 1. Introduction

Fusarium head blight (FHB) is a devastating fungal disease that has been infecting cereal crops, including barley (*Hordeum vulgare* L.), for decades in Canada and worldwide. The main causal species of FHB is *Fusarium graminearum*. There is some variation among host species, but the disease is generally characterized by discoloured, shrivelled, and/or aborted kernels. Of greater concern, however, is the accumulation of mycotoxins like deoxynivalenol (DON) in infected grain and plant tissues [1] due to the deleterious health effects experienced following the consumption of contaminated grains. To malting barley, *Fusarium* infection reduces kernel plumpness and germination and degrades β-amylase, the enzyme responsible for cleaving maltose, the most important fermentable sugar in the beer brewing process [2,3]. Grain with high concentrations of DON is not marketable and requires additional conditioning and processing for sale and safe consumption. FHB affects both grain quantity and quality, affecting growers’ yields, profit, and ultimately their livelihoods.

*Fusarium* species have a very broad range of hosts, mainly comprising graminaceous plant species. Each species of *Fusarium* may infect multiple host species, and multiple *Fusarium* species with overlapping host ranges may concurrently infect the same host plant. For example, *F. graminearum* can cause crown rot in soybean, ear rot in corn, and FHB in wheat, barley, and other small grain cereals, and over 30 different species of *Fusarium* have been detected in *Fusarium*-damaged barley [4,5,6,7,8,9,10,11,12]. Conversely, many species of *Fusarium* can colonize certain host plants but do not present as pathogens (producing typical disease symptoms), like *F. verticillioides* in corn or *F. graminearum* in grassy weeds [13,14]. The *Fusarium* genus itself is highly diverse and each species has nuanced behavior in various hosts [15].

*F. graminearum* is well documented as the dominant causal species of FHB epidemics in Canada [16]. However, in recent years, attention has also been given to *Fusarium poae*, a generally less aggressive pathogen. Multiple surveys have detected *F. poae* in both *Fusarium*-damaged and asymptomatic grain [4,5,11]. This is of concern mainly because *F. poae* produces different mycotoxins, including regulated toxins such as nivalenol that are more toxic than those produced by *F. graminearum*, in addition to other toxins such as beauvericin that are not yet regularly monitored in our feed and food systems [11,17]. Some fungicide applications to control *F. graminearum* are also less effective when *F. poae* is also present [18]. A specific survey of putatively infected grain in Ontario reported that *F. graminearum* was predominant in wheat, *F. poae* was predominant in oats, and in barley both *F. graminearum* and *F. poae* were detected equally as often [11]. The same survey also found that in years where environmental conditions were favorable to *F. graminearum* infection (i.e., warm and humid), *F. graminearum* caused FHB epidemics. This difference in species detection led to the following question: do *F. graminearum* and *F. poae* interact in barley?

The infection models for FHB differ between wheat and barley. In wheat, *F. graminearum* infects the wheat head during anthesis, spreading downward from the point of infection and choking out spikelets above the infection point [19]. In barley, *F. graminearum* infection patterns are different, as hyphae extend across the floret surface and infiltrate through the paleal margins [20]. Mycotoxin production by Fusarium also heavily influences FHB development, with varying effects on disease onset depending on the host species [21]. Field studies on FHB produce complex results due to there being many uncontrolled variables at play, while indoor controlled environment studies help increase understanding of interspecific interactions. The main goal of this study was to compare visual FHB disease, Fusarium biomass, and mycotoxin production in barley when infected with *F. graminearum*, *F. poae*, or both simultaneously. We hypothesized that a competitive interaction exists and that the presence of *F. poae* with *F. graminearum* may increase FHB symptoms, pathogen biomass, and mycotoxin production.

## 2. Materials and Methods

### 2.1. Plant Material

Two susceptible spring barley cultivars, Stander (six-row type) [22] and CDC Bold (two-row type) (CFIA Variety Registration #4951), were tested in growth cabinets at Agriculture and Agri-Food Canada’s Ottawa Research and Development Centre (ORDC) in fall 2021. Seeds were germinated on soaked Whatman paper, and then five seeds per 7 inch pot (The HC Companies, Twinsburg, OH 44087, USA) were transferred to a growth cabinet (Model PGC20, Conviron, Winnipeg, MB, Canada) at 20:17 °C with a photoperiod of 16 h light:8 h dark and 70% relative humidity (RH). Two weeks after planting, plants were fertilized once a week with 20-20-20 N:P:K until harvest.

### 2.2. Inoculum Production

The experiment used one isolate, DAOMC 180378, from *F. graminearum* and one isolate, DAOMC 252242, from *F. poae*. Both isolates were obtained from the Ottawa Valley ecozone and acquired from the Canadian Collection of Fungal Cultures in Ottawa, ON, Canada. The *F. graminearum* strain DAOMC 180378, which is of the 15-ADON chemotype, is frequently utilized in the FHB field nursery at the Ottawa Research and Development Centre for screening FHB resistance in breeding programs [23]. The *F. poae* strain DAOMC 252,242 was selected as it was isolated from cereal crops, showed robust growth in laboratory conditions, and has previously been shown during axenic culturing to exhibit consistent and diverse secondary metabolite/mycotoxin production that is representative for the species [24].

The liquid inoculum (spore suspension) was prepared in the ORDC, Ottawa, ON, as described in Xue, A., K. Armstrong, H. Voldeng, G. Fedak, and C. Babcock [25]. To prepare the culture plates, 0.5 mL of concentrated conidial spore suspension was spread onto modified potato dextrose agar (dextrose, 10 g/L) amended with 20 ppm streptomycin sulfate in 90 mm Petri dishes. Petri dishes were incubated at 22–25 °C under UV and fluorescent lighting for 48 h to stimulate sporulation. Each dish received 10 mL of sterile distilled water with 0.01% Tween 20 (polyoxyethylene sorbitan monolaurate) and was scraped gently with a sterile microscope slide to dislodge spores. The resulting suspension was filtered through two layers of cheesecloth; the spore concentration was determined using a hemocytometer and adjusted to 3.8 × 10^3^ spores/mL. Separate suspensions were prepared for each isolate, and the final concentration of the co-inoculation treatment contained a 1:1 mixture of both species [25].

### 2.3. Inoculation and Scoring Disease Symptoms

The two barley cultivars, Stander and CDC Bold, and four inoculation treatments (*F. graminearum* (*Fg*), *F. poae* (*Fp*), both species simultaneously (*Fg* + *Fp*), and a sterile water control) were arranged in a randomized complete block design with four replications. Spikes were spray-inoculated at 10–14 days after heading [26,27] with spore suspension (approximately 1 × 10^4^ spores per spike) using a kitchen aerosol oil dispenser (Misto International LLC, Bethel, CT, USA), and each head was individually covered with a plastic sample bag for 72 h (Thermo Fisher Scientific, Waltham, MA, USA).

The inoculated pots were transferred to a new growth cabinet with 16 h of light at 25 °C, 8 h of dark at 20 °C, and 90% relative humidity. At 4, 7, 14, 21, and 28 days post-inoculation, a visual disease rating was assigned to each spike on a scale of 0–9 [25]. At 28 days post-inoculation, spikes were cut from the plant at the base of the head, individually wrapped in aluminum foil, flash-frozen in liquid nitrogen, and stored at −80 °C (Thermo Fisher Scientific, Waltham, MA, USA) until ready for further molecular analysis.

Two-way analysis of variance was performed using a mixed procedure of SAS 9.4 to test the differences between the treatments and cultivars and their interaction. Tukey’s honestly significant difference test was performed for the pairwise comparison of means. To achieve a normal distribution, all data were log-transformed. Student’s *t*-test was used to compare the mean value of each treatment with the general mean of all treatments.

### 2.4. Sampling, DNA Extraction, and Quantification of Fungal Biomass

Seven heads (or less if not available) were pooled from each pot and hand-ground as finely as possible in liquid nitrogen using a mortar and pestle. The genomic DNA of each pooled sample was extracted using Macherey-Nagel’s Nucleospin 96 Plant II Kit (Macherey-Nagel, Düren, Germany) with some modifications to the User Manual centrifuge processing protocol. Fungal DNA from in vitro positive controls was isolated using the E.Z.N.A Fungal DNA Mini Kit (Omega Bio-Tek Inc., Norcross, GA, USA) according to the manufacturer’s manual instructions. The concentration of each stock was measured using a NanoDrop 8000 spectrophotometer (Thermo Fisher Scientific, Ottawa, ON, Canada).

### 2.5. Polymerase Chain Reaction (PCR) to Confirm Specificity

The list of species-specific primers used is listed in Table 1. To remove the secondary structure, 1500 ng of extracted genomic DNA was digested with EcoR1 at 37 °C for 60 min. Polymerase chain reaction (PCR) amplification was performed on all digested samples in a 25 μL reaction volume. The final volumes of each reagent were as follows: 0.6 μL of dNTPs, 0.35 μL of 50× Advantage DNA Polymerase, 2.5 μL of 10× Advantage Buffer, 0.6 μL each of forward and reverse primer at 10 μM, 5 μL of template DNA, and 15.35 μL of water. Products were then visualized by gel electrophoresis.

### 2.6. Sanger Sequencing for Amplicon Confirmation

The PCR products were directly transferred and amplified for Sanger sequencing in a reaction volume of 10 μL using the ABI BigDye Terminator 3.1 sequencing kit. The final volumes of each reagent were as follows: 8.5 μL of BigDye Seq Mix diluted 1:8 with Seq buffer (Thermo Fisher Scientific, Ottawa, ON, Canada), 0.5 μL of reverse primer at 3.2 ng/μL, and 1 μL of PCR product from the initial amplification. PCRs were run on an Eppendorf MasterCycler (Thermo Fisher Scientific) with an initial denaturation of 95 °C for 3 min, followed by 40 cycles of 15 s at 95 °C, 15 s of annealing at 50 °C, and 2.5 min of extension at 60 °C, and then a final hold at 10 °C. These PCR products were then submitted for in-house Sanger sequencing. The returned sequence was aligned in Geneious Prime version 2022.1.1 (Biomatters, Ltd., San Diego, CA, USA). The extracted consensus sequence was verified against reference sequences for *Hordeum vulgare*, *F. graminearum*, and *F. poae* using the National Center for Biotechnology Information’s (NCBI) blastn suite as part of their web Basic Local Alignment Search Tool (BLAST) [28].

### 2.7. Droplet Digital PCR (ddPCR) for Fungal Load Quantification

Droplet Digital PCR (ddPCR) conditions were optimized for each reaction by running a series of concentration and temperature gradients with positive and negative controls, and blended test samples. This means when testing *Fg*-specific primers, for example, fungal DNA isolated from pure in vitro culture was the positive control, DNA from barley samples mock-inoculated with ddH_2_O (assumed to be free of fungal infection) was the negative control, and a blend of DNA isolated from *Fg*-treated and co-inoculated barley samples was used to test the efficacy of the primer. *Fg*-treated samples should contain *Fg* DNA, and co-inoculated samples should contain both *Fg* and *Fp* DNA to test *Fg* primer specificity against a background of *Fp* and barley DNA. The final volumes of each reagent were as follows: 12.5 μL of EvaGreen Supermix (Bio-Rad Laboratories Ltd., Mississauga, ON, Canada), 1.6 μL each of forward and reverse primers at 10 μM, 4 μL of digested genomic DNA at 1.25 ng/μL, and 5.3 μL of sterile Milli-Q water, producing a total reaction volume of 25 μL.

A separate ddPCR reaction was performed for each pathogen using species-specific primers with the QX200™ Droplet Digital™ PCR System (Bio-Rad Laboratories Ltd.), meaning the 64 samples were analyzed twice: once with *Fg*-specific primers, and then again with *Fp*-specific primers in a separate reaction. The droplets were prepared with the Bio-Rad Droplet Generator according to the manufacturer’s instructions. PCR amplification was completed on the Bio-Rad C1000 Touch Thermal Cycler with the following conditions: initial denaturation at 95 °C for 5 min, followed by 40 cycles of 95 °C for 30 s and 62 °C for 1 min, then a final hold at 4 °C. Following amplification, the plate was transferred to the Bio-Rad Droplet Reader and droplets were counted with the accompanying software, QuantaSoft version 1.7 (Bio-Rad Laboratories Ltd.). The positive droplet threshold for GRA1 was set manually at 20 K. One control sample (in well C1, barley DNA) was greatly contaminated with GRA1 and so it was removed from the remainder of the analysis. Background noise was detected in the APS1 samples, and this was manually cleaned with thresholds above 22 K and below 28 K.

### 2.8. In Planta Sample Extraction for Metabolomic Analysis

Seven heads (or less if not available) were pooled from each pot, flash frozen, and hand-ground as finely as possible in liquid nitrogen using a mortar and pestle. The hand-ground frozen tissue was then loaded into a ball mill receptacle and milled for 5 min (Retsch MM2000, Newtown, PA, USA). From each milled sample, an allotment of 500–600 mg was transferred into a 10 mL Falcon tube, to which 3 mL of extraction solvent (ACN: H_2_O (4:1; *v*:*v*) with 1% formic acid and 1 ppm of reserpine) was added. Samples were placed in a sonicating bath (FS30D, Fisher Scientific, Waltham, MA, USA) for 5 min, placed on a nutator mixer for 60 min (Medmark Technologies LLC, Perkasie, PA, USA), and then centrifuged for 10 min at 4740× *g* (SORVALL RC-5B Plus, DuPont, Wilmington, DE, USA). Then, 500 μL of the supernatant was transferred into a 2 mL amber vial and submitted for in-house UPLC-HRMS analysis.

### 2.9. Ultra-Performance Liquid Chromatography–High-Resolution Mass Spectrometry (UPLC-HRMS) Analysis

The order of the extracts submitted for UPLC-HRMS analysis was randomized and MeOH blanks were interspaced into the injection order every 5 samples. Chromatography was performed on a Thermo Ultimate 3000 for UPLC using a Phenomenex Kintex C18 column (50 mm × 2.1 mm, 1.7 μm, 100 Å) with a flow rate of 0.35 mL/min and a gradient of two solvents: H_2_O with 0.1% formic acid (*v*/*v*) and acetonitrile (ACN) with 0.1% formic acid (*v*/*v*). The chromatography parameters were as follows: start at 5% ACN and increase to 95% by 4.5 min, hold at 95% ACN until 8 min, return to 5% ACN by 9 min, and hold to equilibrate the column back to starting conditions by 10 min. HRMS detection was performed using an LTQ OrbitrapXL mass spectrometer (Thermo Fisher Scientific; Waltham, MA, USA). The machine was used with the following acquisition parameters: positive electrospray ionization (ESI+) mode, range of 100–2000 *m*/*z*, resolution of 30 K (at 400 *m*/*z*), automatic gain control target of 5 × 10^5^, and maximum injection time of 500 ms; and source parameters: ionization voltage of 4.0 kV, capillary voltage of 34 V, tube lens voltage of 100 V, sheath gas of 40, auxiliary gas of 5, and sweep gas of 2.

### 2.10. UPLC-HRMS Data Curation and Targeted Metabolomics Analysis

UPLC-HRMS .raw files were processed through mzMine v2.53 [29] prior to performing metabolomics. Mass features were detected with a noise level threshold of 1.0 × 10^4^. Chromatograms were built using the ADAP chromatogram builder with a minimum group size of 7 scans, group intensity threshold of 1.0 × 10^5^, minimum highest intensity of 1.0 × 10^6^, and *m*/*z* tolerance of 0.001 *m*/*z* or 5.0 ppm. Peaks were smoothed with default settings and then de-convoluted using the baseline cut-off algorithm where the minimum peak height was set to 1 × 10^6^ with a baseline level of 1 × 10^5^. Chromatograms were aligned with the join aligner; the *m*/*z* tolerance was 0.001 *m*/*z* or 5.0 ppm, the weight of *m*/*z* was 2, the retention time (RT) was 0.2 min, and the weight of RT was 1. Once gap-filled (*m*/*z* tolerance of 0.001 or 5.0 ppm), data were exported to Microsoft Excel for further curation, in which a short list of mass features that were present in in planta pathogen-challenged plants and absent in mock-infected controls (representing fungal secondary metabolite production or other unknown (fungal or plant) metabolites associated with infection) were carried forward for metabolite annotation.

Mass features were annotated based on an accurate mass (<5 ppm) comparison to an in-house database of *F. graminearum* and *F. poae* secondary metabolites. To substantiate mass feature annotations, manual inspection of the raw MS data was performed in the QualBrowser package in ThermoXcalibur 2.2 SP1.48 to confirm the assigned associated mass feature adduct and neutral loss state assignments and to compare the observed RT and *m*/*z* information to internal standards when possible. All annotated mass features were carried forward as a data matrix for targeted metabolomic analysis. Mass feature peak heights in the targeted data matrix were normalized to the total ion current (TIC) of each sample. Multivariate and univariate statistical analysis of the resulting data matrix was completed using the Metabolomics Univariate and Multivariate Analysis (muma) package [30]. The one-factor statistics module of MetaboAnalyst v5.0 was used to generate heat maps of auto-scaled mass feature intensities across samples and to perform analysis of variance (ANOVA) of selected mass features.

## 3. Results

### 3.1. Effect of Fusarium spp. On FHB Symptoms

**Figure 1:**
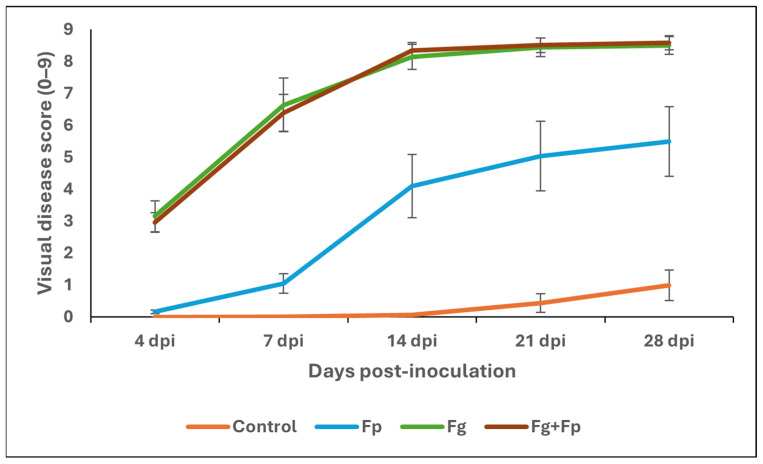
Mean observed Fusarium head blight (FHB) disease severity on Stander and CDC Bold barley cultivars in the growth chamber experiment. The disease was assessed as described by Xue et al. [25].

Two-way ANOVA was used to analyze the effects of barley cultivars and fungal species inoculation treatments on visual FHB symptoms (Table 1). There was a significant treatment effect at all time points (*p* < 0.0001), while the cultivar genotype effect was significant (*p* < 0.05) only between 4 and 7 dpi. In early disease progression, *Fp* alone was not significantly different from the control treatment, but the FHB severity was significant at 14 days post-inoculation and beyond. The FHB ratings from *Fp* alone were significantly lower than the FHB ratings from *Fg* and co-inoculation (*Fg* + *Fp*) (Figure 1).

### 3.2. Fusarium graminearum and Fusarium poae DNA Quantification

**Figure 2:**
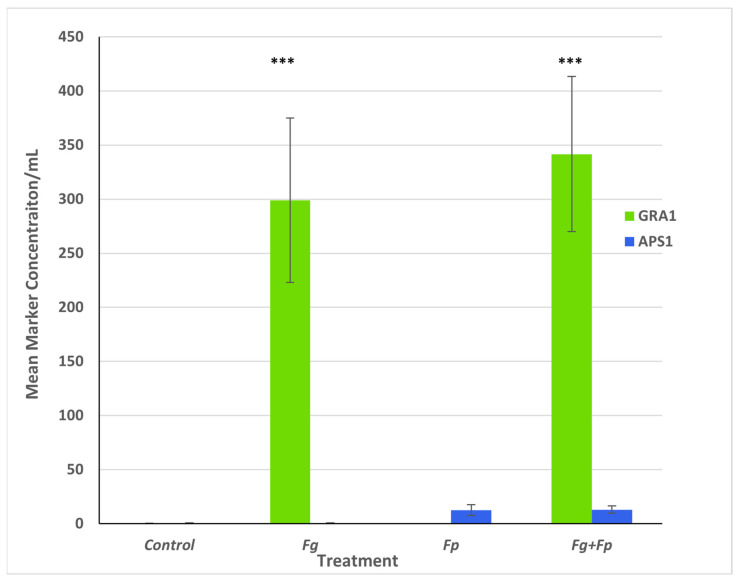
Gene expression of the *Fusarium* secondary metabolite biosynthetic genes *GRA1* and *APS1* in infected barley spikes. Expression was measured by ddPCR on ground tissue inoculated with single-species or co-inoculation treatments. Heads were harvested at 28 days post-inoculation (dpi). *** *p* < 0.0001. Error bars represent standard error of mean.

To compare the response to the different inocula using a DNA marker gene, the ratio of the *GRA1* gene to the *APS1* gene was used. Multiple ANOVA was used to analyze the effects of cultivar, treatment, and replicate on the ratio of mean *GRA1* concentration to mean *APS1* concentration; of the comparators used, only treatment had a statistically significant effect (*p* < 0.05). The ratios of *GRA1* to *APS1* between *Fg*-treated and co-inoculated (*Fg* + *Fp*) samples were similar; that is, the *GRA1*-to-*APS1* ratio in samples treated with *Fp* alone was significantly lower than in the *Fg*-treatment and co-inoculated samples (*Fg* + *Fp*) (Figure 2).

### 3.3. Targeted Metabolomics of in Planta Challenge Experiments

A total of 36 detected mass features from the in planta pathogen challenge experiments were assigned putative names with associated production from either *F. graminearum* or *F. poae*. In both monoculture and co-culture in planta trials, *F. graminearum* was observed to produce deoxynivalenol (DON), 15-acetyldeoxynivalenol (15-ADON) (and numerous other associated trichothecene biosynthesis intermediate products), fusaoctaxins (A and B, fusapentaxin B, and other analogs), culmorin, zearalenone (ZEA), and fusaristatin hydroxyrubrofusarin-fuscofusarin. The manual examination of UPLC-HRMS data files also confirmed the production of gramillins and fungal decalin-containing diterpenoid pyrones (FDDPs); however, associated mass features were excluded from the metabolomic analysis due to the chosen threshold parameters during metabolomics data preprocessing. From the monoculture and co-culture in planta challenges using *F. poae*, mass features associated with several cyclic peptides were observed, which included several apicidins, beauvericin (BEA), and W493-B. Despite being a known producer of both type A and B trichothecenes (i.e., nivalenol, diacetoxyscirpenol, fusarenon X, etc.), no trichothecene metabolites were observed to be produced in planta by *F. poae*.

**Figure 3:**
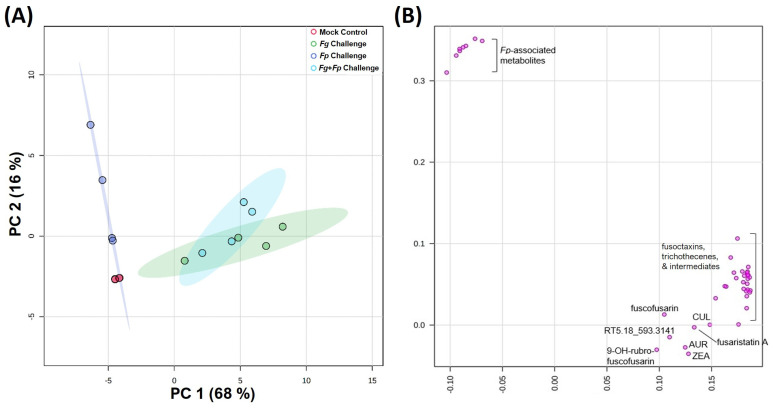
Results from PCA analysis of barley cultivar Stander, comparing *F. graminearum* and *F. poae* secondary metabolite mass feature associations between monoculture and co-culture pathogen challenges. (**A**) PC1-2 score plot (red dots = mock control samples; green dots = *F. graminearum* monoculture samples; dark-blue dots = *F. poae* monoculture samples; light-blue dots = *F. graminearum* and *F. poae* co-culture samples). (**B**) PC1-2 loading plot representing mass feature variables (*Fp* = *F. poae*).

To compare the production of pathogen-associated secondary metabolites (mycotoxins) within the different in planta experiments (monoculture vs. co-culture and mock infection controls), principal component analysis (PCA) was performed on individual barley cultivar datasets. The PCA models for both cultivars were comparable in terms of observed trends, with both models optimizing after two PCs, explaining 84% of the variance in the data model for both Stander (Figure 3) and CDC Bold. In both PCA models, there was a clear separation of monoculture samples along PC1 (explaining 68% of the variance in the Stander model and 69% of the variance in the CDC Bold model), with *F. poae* monocultures and mock infection controls separating from the *F. graminearum* monoculture and *F. graminearum*/*F. poae* co-culture samples along PC1 (Figure 3A). PC2, which explained 16% of the variance in the Stander model and 15% of the variance in the CDC Bold model, separated samples based on the presence of *F. poae* metabolites (as observed from PCA loading plots; Figure 3B), with a positive directional movement of *F. poae* monoculture samples from the mock-infected controls and *F. graminerum*/*F. poae* co-culture samples from the *F. graminearum* monoculture samples along PC2 (Figure 3A). Confidence ellipses (95%) drawn on the PCA score plot were used to demonstrate the overlap boundaries between the sample groups in the data model. There was a distinct overlap of *F. graminearum* monoculture and *F. graminearum*/*F. poae* co-culture confidence ellipses due to the dominance of *F. graminearum* metabolites within the samples from both classes (as observed in loadings plots; Figure 3B).

**Figure 4:**
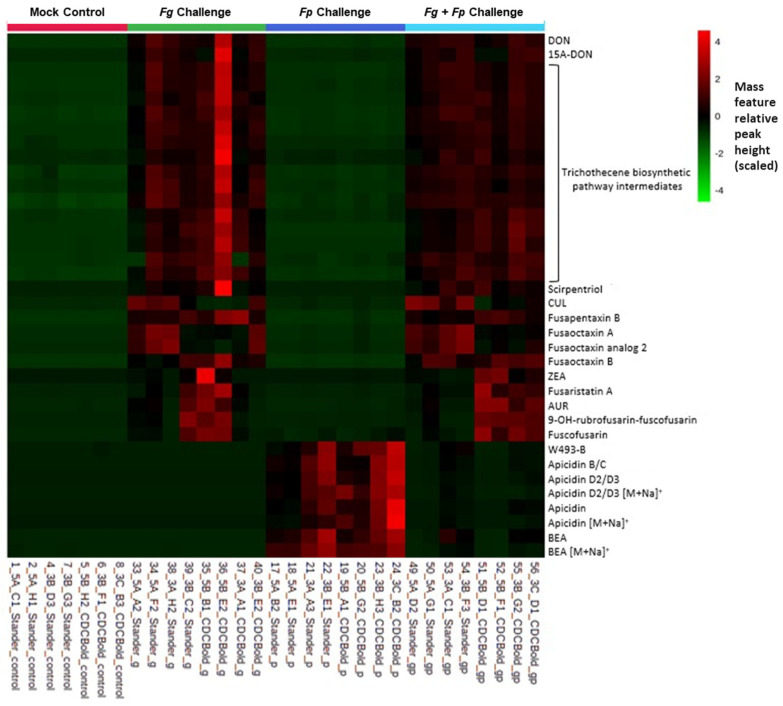
Heatmap demonstrating difference in scaled (4 to −4) relative abundance of mass feature intensities observed in in planta challenge experiments using Stander and CDC Bold barley cultivars (red colour reflects greater abundance; black reflects low abundance; green reflects absence). Protonated pseudomolecular ion ([M + H]^+^) mass features are used to represent the various metabolites, unless otherwise specified.

A heatmap of mass feature relative abundance was constructed using scaled intensities to view the distribution of metabolite production between the various in planta challenge samples (Figure 4). As was observed from the PCA models, both *F. graminearum* and *F. poae* monoculture samples had distinct and different secondary metabolite compositions. Metabolite production was comparable with only minor variations observed between Stander and CDC Bold cultivars in the various in planta challenge samples. In the *F. graminearum*/*F. poae* co-culture challenge experiments, *F. graminearum*-associated secondary metabolites were more abundant in infected tissues as compared to *F. poae* metabolites. The observed differences in secondary metabolite relative abundance reflected the observed trends from the PCA modeling, in that some differences were observed between sample replicates, especially in terms of the relative abundance from *F. poae*-associated metabolites (in both monoculture and co-culture samples). As was noted earlier, no evidence was observed of trichothecene production in the *F. poae* monoculture challenge in either barley cultivar, nor was a discernable increase in trichothecene production (in terms of relative abundance) observed between *F. graminearum* monoculture samples and *F. graminearum*/*F. poae* co-culture samples (except a single *F. graminearum* sample replicate in CDC Bold).

## 4. Discussion

Understanding the interactions of *Fusarium* spp. may facilitate improvement in FHB control strategies. In most published studies on pathogen interaction, competition is the norm rather than the exception [31], and our results suggest that the competition was biased, despite the fact that plants were grown at the optimal temperature for *F. poae* (25 °C) rather than the optimal 28 °C for *F. graminearum*. *F. graminearum* has been and continues to be the primary cause of FHB epidemics in cereal crops across Canada [32]. In fact, its range seems to be expanding, as it is also becoming the dominant cause of epidemics in cooler regions of Europe [11,33]. There has been extensive research into what makes *F. graminearum* so potent, but there is no single straightforward reason. Most studies focus on examining the genetic regulation of *F. graminearum*’s attack (and subsequent defense by the host to develop resistant cultivars), as well as differences in mycotoxin production.

Numerous disease surveys and pathogenicity tests in controlled environments and the field have reported the dominance of *F. graminearum* in causing FHB [10,34,35]. In one study, Brennan, J., B. Fagan, A. Van Maanen, B. Cooke, and F. Doohan [36] cultured five different *Fusarium* species in vitro and conducted in planta pathogenicity tests in wheat coleoptiles. *F. graminearum* and *F. culmorum* were the most pathogenic and damaged coleoptiles nearly twice as severely as *F. poae*. Indoor experiments by Xue, A. G., K. M. Ho, G. Butler, B. J. Vigier, and C. Babcock [27] examined the pathogenicity of eight species of *Fusarium* in barley under controlled conditions. They inoculated six cultivars with 48 isolates 10–14 days after heading, and also recorded *F. graminearum* as highly pathogenic and *F. poae* as weakly pathogenic. A different study by Xue, A., K. Armstrong, H. Voldeng, G. Fedak, and C. Babcock [25] on wheat reported similar findings: six wheat lines were inoculated with eight species of *Fusarium* (54 isolates), and *F. graminearum* not only produced the most severe disease symptoms, but also produced the most severe symptoms most rapidly.

When co-inoculated with multiple *Fusarium* sp. in one system, it was expected that we would observe interspecific competition. From our observations, *F. poae* does not appear to compete with *F. graminearum* when inoculated indoors. Xu et al. [37] inoculated wheat heads with one of four different *Fusarium* species (*F. graminearum*, *F. culmorum*, *F. avenaceum*, and *F. poae*), varying initial inoculation conditions. They then inoculated the samples up to 48 h later with another two or three of the listed species. The authors reported that inoculation with additional species left wheat heads no more severely changed than single-species inoculation and did not significantly affect visual disease ratings. We observed similar behavior in our experiments, where co-inoculation did not dramatically change visual disease ratings; the disease severity observed in our trials appeared relatively unchanged when heads were co-inoculated with *F. graminearum* and *F. poae*, as compared to *F. graminearum* alone.

To investigate fungal interactions, many groups have used mixed co-inoculations where spores from both species are in the same inoculum suspension, as we did in our work, and/or have used sequential inoculations where spores from both species are applied separately in different time periods. Where there is a difference in aggressiveness in the pathogens, the more aggressive pathogen generally isolates more fungal DNA [37]. Visual disease ratings from inoculation with both *F. graminearum* and *F. poae* looked no different than barley spikes inoculated with *F. graminearum* alone, where at 28 days post-inoculation, the mean visual disease rating was 8.5 out of 9 when inoculated with *F. graminearum* alone, and 8.58 out of 9 when co-inoculated with both *F. graminearum* and *F. poae*. By contrast, the disease ratings of *F. poae* inoculated singly were much lower at 5.5 out of 9. Taken together, this evidence supports the hypothesis that *F. poae* is either a “weak pathogen” or possibly a non-antagonistic endophyte, and that *F. poae* does not antagonize *F. graminearum* in the parameters of this study.

The ddPCR results show that the level of *GRA1* found in barley heads remains the same when *F. graminearum* is inoculated with *F. poae* compared to when *F. graminearum* is inoculated alone. It was expected that *F. graminearum* would be the dominant species as the disease ratings were similar regardless of whether *F. poae* was present during the inoculation. The dominant pathogen, *F. graminearum*, did not gain a selective advantage when co-inoculated with the weaker *F. poae*. However, colonization by the weaker pathogen, *F. poae*, did not appear to be reduced by the more aggressive *F. graminearum*. The fungal DNA accumulation of both species did not seem to be related. This is unusual compared to other studies where *F. graminearum* and/or *F. poae* biomass are reduced in the presence of a competitor [37,38,39].

In a co-inoculation of *F. avenaceum* and *F. poae*, the amount of *F. poae* DNA was lower than when inoculated alone, which suggests that *F. avenaceum* suppressed or antagonized *F. poae* [37]). However, the accumulated levels of nivalenol (NIV), a toxin reported only from *F. poae* and not *F. avenaceum*, were nearly 1500 times higher in co-inoculation than in *F. poae* alone. This suggests that the NIV productivity per unit of fungal biomass is dramatically increased in co-inoculation compared to single-species inoculation, and that mycotoxin accumulation is another important parameter that should be investigated to understand the effect of *Fusarium* spp. interactions on grain quality.

Many research groups have seen that mycotoxin accumulation increases in co-inoculations when fungi are assumed to be competing for resources [40]. Xu, X. M., W. Monger, A. Ritieni, and P. Nicholson [37] observed that mycotoxin accumulation levels were up to 1000 times greater in wheat when *F. graminearum* was co-inoculated with *F. culmorum* and *F. poae* than in single-species inoculations. Simpson, M., N. Taylor, and K. Barker [38] investigated the in vitro mycotoxin production of *F. culmorum* and *Microdochium majus* over time. They initially saw that pure *F. culmorum* and mixed cultures produced similar levels of DON, despite reduced *F. culmorum* fungal biomass from competition with *M. majus*, and suggested that DON productivity per unit of fungal biomass was higher in response to competition. We observed no increases in DON production when *F. graminearum* was cultured with *F. poae* in barley.

When barley was co-inoculated with *F. graminearum* and *F. poae*, the observed mycotoxin profile closely resembled the individual *F. graminearum* treatment. The disease was more intense in plants infected with *F. graminearum* (simultaneously with *F. poae* or not), and more *F. graminearum* genomic DNA was isolated than *F. poae*. However, the levels of *F. graminearum*-associated metabolites in the co-inoculation were similar to those reported in the individual *F. graminearum* treatment. Moreover, levels of *F. poae*-associated metabolites were significantly lower in the co-inoculation. Notably, the trichothecenes detected were exclusively associated with *F. graminearum* trichothecene profiles. This evidence is consistent with the hypothesis that *F. graminearum* outcompeted *F. poae* within the parameters of the co-inoculation experiment.

In summary, when barley was challenged with *F. graminearum*, *F. poae*, or both in a controlled environment under high-humidity conditions, it was observed that *F. graminearum* outcompetes *F. poae*. The disease severity was the highest when barley was inoculated with *F. graminearum* alone, and the addition of *F. poae* did not significantly change the disease ratings. Further analysis of the relative amounts of *F. graminearum* and *F. poae* revealed that the ratio of genomic DNA of *F. graminearum* to *F. poae* remained consistent whether samples were inoculated with both *F. graminearum* and *F. poae* or with either species alone. This indicated that *F. poae* did not affect *F. graminearum*’s ability to colonize barley, and vice versa. The amount of genomic DNA from *F. graminearum* was greater than that of *F. poae*, indicating that *F. graminearum* was more successful in colonizing barley spikes under the conditions used in this study. Metabolomic analysis showed that *F. graminearum*-associated metabolites dominated the mycotoxin profile of co-inoculated samples, suggesting that treatment with *F. graminearum* significantly influenced the mycotoxins accumulated in barley grain when challenged with both species. Although fungal metabolism varied slightly between barley cultivars, further studies with more cultivars of varying FHB resistance levels are needed to explore this observation in more detail. A previous study [11] has reported that *F. poae* detection is greater in non-epidemic conditions, indicating a need for further investigation under different growth conditions to test whether individual species’ fitness is affected. The use of sequential inoculations rather than mixed inoculations, as performed in other fungal interaction studies with *Fusarium* spp., would be valuable in observing the competition between *F. graminearum* and *F. poae*. Most research is focused on protecting barley from *F. graminearum*. If resistant varieties behave similarly between *F. graminearum* and *F. Poae*, i.e., they are relatively unaffected by *F. poae*, then breeders can feel more confident in selecting resistant cultivars for barley growers. However, this needs to be tested with more *F. graminearum* and *F. poae* isolates. It is also important for future investigations to consider more species of *Fusarium* beyond *F. graminearum* and *F. poae*, as these are not the only FHB-causing species relevant to farmers.

## Figures and Tables

**Table 1:** Two-way ANOVA mean square values for genotype and treatment effects from growth cabinet once inoculated with *F. graminearum*, *F. poae*, or both.

Source	Df ^1^	4 dpi ^2^	7 dpi	14 dpi	21 dpi	28 dpi
Genotype	1	2.65 *	19.69 **	4.43	3.45	1.09
Treatment	3	23.68 ***	96.92 ***	123.11 ***	116.82 ***	101.08 ***
Genotype × Treatment	3	1.02	6.46	0.97	1.14	2.97

^1^ df = degrees of freedom. ^2^ dpi = days post-inoculation. *, **, ***: significant at *p* < 0.05, *p* < 0.001, *p* < 0.0001, respectively.

## Data Availability

The original contributions presented in the study are included in the article, further inquiries can be directed to the corresponding author.
